# Ultra-Violet Protection and Anti-Static Characteristics with Heat Release/Shielding of Al_2_O_3_/ATO/TiO_2_-Imbedded Multi-Functional Fabrics

**DOI:** 10.3390/ma15103652

**Published:** 2022-05-20

**Authors:** Hyun-Ah Kim

**Affiliations:** Korea Research Institute for Fashion Industry, Daegu 41028, Korea; ktufl@krifi.re.kr

**Keywords:** UV protection, anti-static, FIR, heat shielding, SER

## Abstract

This study examined the ultra-violet (UV) protection and anti-static characteristics with heat release by far-infrared (FIR) emissivity of Al_2_O_3_/Antimony Tin Oxide (ATO)/TiO_2_-imbedded PET fabrics according to the weight (wt.) percentage of the Al_2_O_3_/ATO particles imbedded in the yarns. The fabric with lower Al_2_O_3_/ATO wt. percentage had more effective heat release than that of the fabric with higher percentage due to the heat shielding property of the ATO particles. The fabric with lower Al_2_O_3_/ATO wt. percentage (higher TiO_2_) exhibited higher UV protection factor (UPF) than that of the fabric with higher percentage, which suggested that the UV protection characteristic of TiO_2_ particles imbedded in the yarn was superior to that of the Al_2_O_3_/ATO particles. The anti-static property of the higher wt.% of ATO-imbedded fabric was superior to that of the lower wt.% of ATO-imbedded fabric, which means that ATO inorganic particles provide superior anti-static property to the Al_2_O_3_ and TiO_2_ particles. On the other hand, Al_2_O_3_/ATO particles imbedded in the yarns imparted an uncomfortable tactile hand feel and wear fitness to the fabrics.

## 1. Introduction

Of the many studies that have recently explored the application of inorganic particles to textile goods, some studies [1,2,3,4,5,6,7,8,9,10] on the heat release and storage characteristics using ZrC, Al_2_O_3_ and TiO_2_-incorporated PET fabrics have been conducted by some scientists [1,2,3,4,5,6] and Japanese companies [7,8,9,10] for a couple of decades. Recently, Kim and Kim [4,5,6] examined the thermal property with the far-infrared emission characteristics and the wear comfort property of the ZrC/Al_2_O_3_/graphite-imbedded PET fabrics. On the other hand, although sunlight is essential to all forms of life on earth, it can also be harmful, particularly exposure to certain components of its radiation, for example, ultraviolet (UV) radiation, this being harmful to both humans and textiles [11]. Consequently, many studies [12,13,14,15,16,17,18,19] were performed to improve UV protection of fabrics using inorganic particles; as a result, it was known that many inorganic particles, such as TiO_2_, ZnO, Al_2_O_3_ and, SiO_2,_ can be applied as UV protection agents [12]. Among them, TiO_2_ [13,14,15,16] and ZnO [17,18,19] are commonly used as UV blocker agents, and their nanoparticles (NPs) are more efficient at absorbing and scattering UV radiation than the conventional particle sizes. However, previous studies [13,14,15,16] increased the effectiveness of UV protection by the coating treatment of TiO_2_ nanoparticles (NPs) on the fabric surface. Other studies [17,18,19] were carried out using the ZnO-coated NPs to obtain more efficient UV protection than the conventional-sized particles, which also used the coating method in their studies. On the other hand, one of the problems generally faced while using wholly synthetic textiles is their tendency to generate static electricity. As synthetic fibers have poor anti-static properties, research work concerning the improvement of the anti-static properties of textiles by using nanotechnology has been undertaken [20]. According to the previous studies [21,22], TiO_2_, ZnO and Antimony Tin Oxide (ATO) particles provide an anti-static property because of their electrically conductive characteristics, i.e., they help to effectively dissipate the static charge accumulated on the fabric. Dong and Huang [21] reported the improvement of the anti-static property of the nano polypropylene/TiO_2_ composite fiber. Wu et al. [22] investigated the anti-static effect and application of the novel fabric finishing agent containing ATO NPs. In particular, out of many types of inorganic particles, ATO exhibits the thermal insulation, heat absorbing and shielding property, and good electric conductivity with environmentally friendly properties [12]. According to previous studies [23,24,25], Ahn et al. [23] examined the thermal insulation property of the ATO-coated glass sheet applicable to the green energy industry. Sun et al. [24] reported the excellent electric conductivity of the ATO-coated PET film applicable to the anti-static electrical field. In addition, Muller et al. [25] reported the high conducting property of the monodispersed ATO NPs synthesized by nonaqueous sol-gel method. However, each of these previous studies investigated the thermal insulation, UV protection and electric conductivity of the inorganic-particle-coated sheets, not inorganic-particle-imbedded yarn and fabric materials, moreover, which was performed using only one type of inorganic-particle-treated specimen. Furthermore, few studies have investigated the UV protection and anti-static characteristics with the emissivity of the far-infrared (FIR) radiation of the Al_2_O_3_, ATO and TiO_2_-imbedded PET yarns and their fabrics, not coated ones. In particular, multi-functional fabrics for cold weather and UV protective clothing are required during winter mountain climbing and in the cold-weather countries. In addition, heat release, anti-static and UV cut characteristics of the multi-functional fabrics have to be maintained with repeated washing and laundering, i.e., a novel method for this, not a coating method, is needed. Therefore, this study examined the UV cut and anti-static properties with heat release by FIR emissivity of the Al_2_O_3_/ATO/TiO_2_-imbedded PET fabrics to develop multi-functional textile materials for high-performance clothing. For this purpose, the Al_2_O_3_/ATO/TiO_2_-imbedded PET yarns with different weight percentages were spun on a conjugated melt spinning machine, which is a novel method (free of washing and laundering), and their fabric specimens were made using three types of Al_2_O_3_/ATO/TiO_2_-imbedded sheath/core yarn, and a TiO_2_-imbedded regular PET fabric specimen was prepared as a control fabric.

## 2. Materials and Methods

### 2.1. Spinning of Yarn Specimens

**Figure 1:**
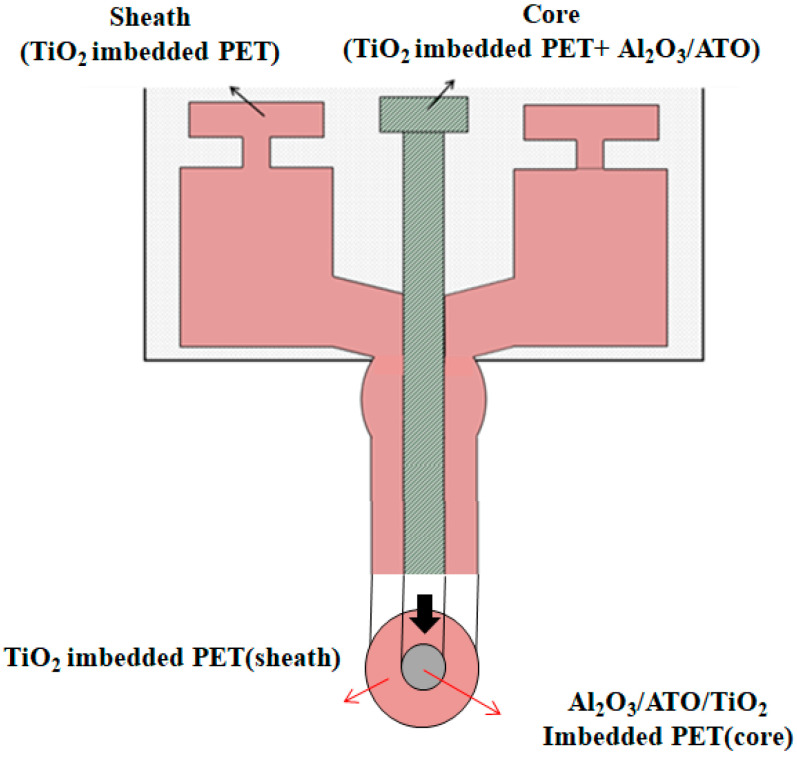
Schematic diagram of the bi-component melt spinning machine.

Three types of Al_2_O_3_/ATO/TiO_2_-imbedded PET yarns were prepared on a bi-component melt spinning machine (TMT Co. Ltd., Kyoto, Japan), which is shown in Figure 1. Figure 1 shows a schematic diagram of the sheath/core typed bi-component melt spinning machine used in this study. Before yarn spinning, first, Al_2_O_3_ and ATO master batch (M/B) chips were made using the mixed polymers combined with 20 wt.% Al_2_O_3_ and ATO particles each and 80 wt.% PET chips on the compounding machine (SM Platek Co., Ltd., Ansan, Korea). Both Al_2_O_3_ and ATO M/B chips were combined with a mixing ratio of 0.8 wt.% Al_2_O_3_ (2 kg) and 0.3 wt.% ATO (0.75 kg), which was mixed with TiO_2-_imbedded PET base polymer (47.25 kg) in the core part in the bi-component melt spinning machine. The mixing ratio of the M/B chip to make Al_2_O_3_/ATO sheath/core PET yarn specimens is presented in Table 1.

Three Al_2_O_3_/ATO/TiO_2_-imbedded sheath/core yarn specimens were spun with PET base polymer (50, 40 and 30 wt.%) in the sheath part and Al_2_O_3_ and ATO-imbedded PET polymer (50, 60, 70 wt.%) in the core part on a bi-component spinning machine, as shown in Figure 1. The bi-component spinning machine was equipped with a 24-hole spinneret with a capillary diameter of 0.24 mm and a length of 0.5 mm. The detailed spinning conditions in the bi-component spinning machine are shown in Table 2. Cross-sections of yarn specimens were measured to find out the inorganic particles imbedded in the yarns using SEM (S-4300, Hitachi Co., Kyoto, Japan).

### 2.2. Preparation of Fabric Specimens

The fabric specimens were woven on the water jet loom (ZW-315X, Tsudakoma, Japan) using three Al_2_O_3_/ATO/TiO_2_-imbedded sheath/core yarns and a TiO_2_-imbedded regular PET yarn as a control yarn with a fixed warp yarn of PET 50d/72f. Table 3 lists the specifications of the four fabric specimens. The woven fabric specimens were scoured in a CPB scouring machine, washed and dyed in a rapid machine and finally washed and dried in a tenter machine.

### 2.3. Elemental Analysis and FIR Measurement of the Yarn Specimens

Elemental analysis of the yarn specimens was performed to verify the Al_2_O_3_/ATO particles imbedded in the yarn using energy dispersive X-ray spectroscopy (EDS: Jeol LV 8500, Tokyo, Japan). The FIR emission experiment for yarn specimens was carried out to estimate the far-infrared ray emitted from the Al_2_O_3_/ATO particles imbedded in the yarn specimens using a Fourier transform infrared (FT-IR) spectrometer (Midac M 2400-C, Irvine, CA, USA). The emissivity and emissive power were measured at 40 °C and over a wavelength range of 5–20 µm. Their mean and deviation for five readings of experimental data were calculated, and the unit for emissive power was W/m^2^∙µm.

### 2.4. Light Heat Emission Measurement

**Figure 2:**
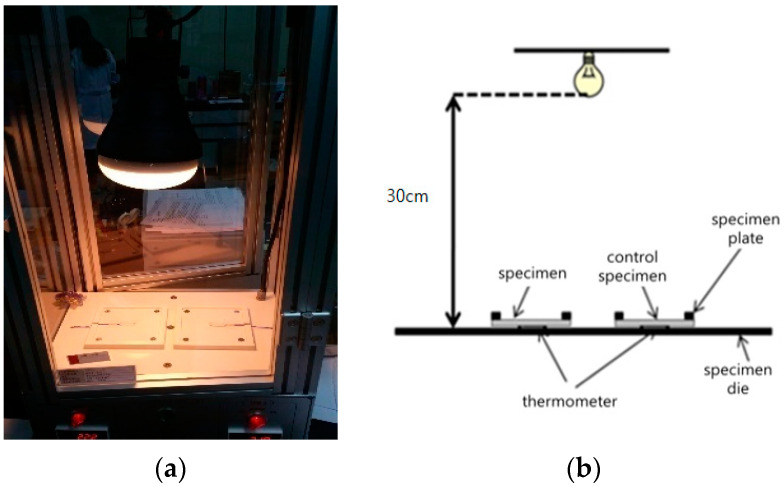
Light heat emission apparatus ((**a**) apparatus, (**b**) schematic diagram).

The thermal radiation of the fabrics was assessed to measure the temperature increment by the heat emitted from the Al_2_O_3_/ATO particles in the yarns using a light heat emission apparatus. Figure 2 shows the light heat emission apparatus used in this study. A specimen sized 10 cm × 10 cm was prepared at a temperature of 20 ± 2 °C and a relative humidity of 65 ± 5 % RH and placed on a thermometer in the specimen die. A heat emission bulb placed 30 cm away from the specimen was switched on, and the temperature change, according to measuring time, was measured and recorded to detect the heat release property of the far-infrared ray emitted from the Al_2_O_3_/ATO-imbedded yarns and their fabrics.

### 2.5. Measurement of UV Protection

The UV protection characteristics of the fabric specimens were measured in the range of 290–400 nm using an ATLAS M 284D SDL equipment, South Carolina, USA. The fabric specimens were conditioned at 20 °C and 65% RH for 24 h. Four scans were obtained during the measurement by rotating the specimens 90° each time, and the spectral data were recorded as the average value of these four scans. The UPF, which is divided into UVA and UVB transmittances, was calculated using Equation (1) [13].

(1) $$\mathrm{UPF}=\frac{\sum_{\lambda =290}^{400}E\left(\lambda \right)\varepsilon \left(\lambda \right)\Delta \left(\lambda \right)}{\sum_{\lambda =290}^{400}E\left(\lambda \right)T\left(\lambda \right)\varepsilon \left(\lambda \right)\Delta \left(\lambda \right)}$$

where E(λ) is the solar irradiance (Wm^−2^mm^−1^) measured, ε(λ) the erythematic action spectrum, Δ(λ) the wavelength interval of the measurements, and T(λ) the spectral transmittance at wavelength λ.

### 2.6. Anti-Static and Electric Resistance Measurement

To examine the electric conductivity of the Al_2_O_3_/ATO/TiO_2_-imbedded yarns and fabrics, two measurements were taken: anti-static and electric resistance. As an anti-static measurement, the static electricity by rubbing of the fabric specimens was measured using the JIS L 1094 standard method [26]. The specimens were pre-dried at 70 °C for 1 h and then conditioned at 20 ± 2 °C and a R.H. of 40 ± 2% for 24 h. Three specimens, 4 cm long and 8 cm wide, were prepared for the four woven fabric specimens. The twenty cotton and worsted rubbing fabrics attached to the measuring apparatus were prepared with a length and width of 16 cm and 2.5 cm, respectively. The static voltage (V) was measured after a drum revolution as a measure of the static electricity, which was conducted for 60 s. The surface electrical resistivity (SER, ρ, Ω/sq) of the fabric specimens was measured to determine the electric resistance using an electric resistance measuring equipment (ACL 800 Megohmmeter, Keithley Instrument Inc., Solon, OH, USA). SER stands for the ratio of applied voltage (volt/meter) to the current (amp/meter) passing through a material, and its unit is ohms per square (Ω/sq = Ω/□). A four-point typed probe is usually used to measure SER, and the correction factor (CF) is multiplicated to calculate surface resistivity as a unit, ohm/sq. Anti-static materials, such as fabric and film with electrical resistivity above 10^7^ ohm/sq, are measured using high voltage in measuring equipment, and in this case, a two-point typed probe or circle typed probe is used instead of a four-point typed probe. In this study, circle typed probe was used with correction factor (2.73), which was calculated considering specimen size, thickness and temperature during experiment. The surface electrical resistivity was calculated using Equation (2) [27].

(2) $$\rho =2.73\times \;R/\log(\frac{r_{o}}{r_{i}})\;\left(\mathrm{ohm}/\mathrm{sq}\right)$$

where 2.73 is the constant, R(Ω) the measured surface electrical resistivity of the specimen, r_i_ the inner radius of the electrode, and r_o_ the outer radius of the electrode.

### 2.7. Measurement of Fabric Mechanical Property

The mechanical properties of the fabric specimens were measured using a Fabric Assurance Simple Testing (FAST) system [28]. Compressibility was measured using a FAST-1 measuring apparatus. Surface thickness (ST, mm), as a measure of compressibility, was calculated as the difference in the thickness of a fabric at a compression of 1.96 cN/cm^2^ and 98.04 cN/cm^2^. Bending rigidity (B, μN·m) was calculated using C, as shown in Equation (3) [28], which was measured using a FAST-2 measuring device.

(3) $$B=W\;\times {\;C}^{3}\times 9.52\times 10^{-6}$$

where C is the bending length (mm), and W is the weight per unit area of fabric (cN/m^2^). The extensibility (E100, %) was measured at a load of 98.04 cN/cm using FAST-3. The shear modulus (G, N/m) was calculated using EB5, as shown in Equation (4) [28], which was measured using a FAST-3 measuring device.

(4) $$G=\left(\frac{123}{\mathrm{EB}5}\right)\times 1\frac{N}{m}$$

where EB5 is the bias extension under a 4.85 cN/cm width in %.

## 3. Results and Discussion

### 3.1. Elemental Analysis and Heat Release Characteristics of the Al_2_O_3_/ATO/TiO_2_-Imbedded Fabrics

**Figure 3:**
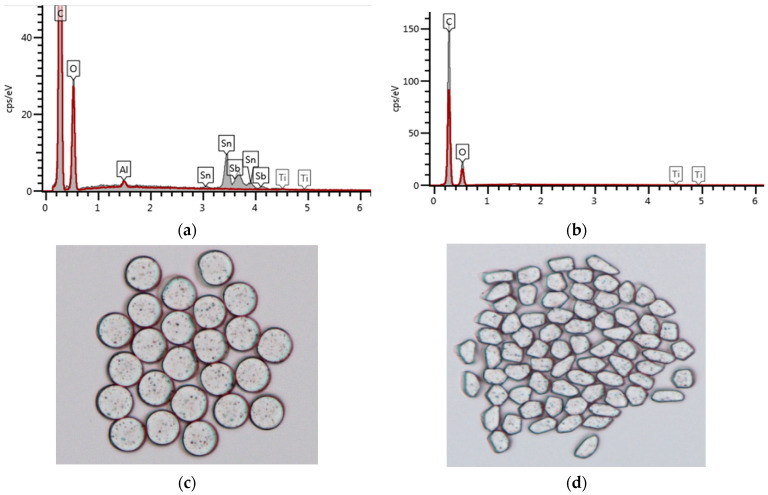
Elemental analysis and microscope images of the Al_2_O_3_/ATO-imbedded and regular PET yarns ((**a**,**c**) Al_2_O_3_/ATO/TiO_2_-imbedded yarn, (**b**,**d**) TiO_2_-imbedded regular yarn).

Before examining the thermal radiation of the fabric specimens, inorganic particles imbedded in the yarns (Al_2_O_3_/ATO/TiO_2_-imbedded yarn and TiO_2_-imbedded regular yarn) were verified by elemental analysis using an EDS. Figure 3a,b present the elemental analysis results of the Al_2_O_3_/ATO/TiO_2_-imbedded (S/C:50/50) and TiO_2_-imbedded regular PET yarns. In Figure 3a, Al, Sn and Sb peaked as the metal ingredient appeared, and Ti was also observed. In addition, Ti ingredient was also observed in the regular PET yarn of Figure 3b. Figure 3c,d show the yarn cross-sections of the Al_2_O_3_/ATO/TiO_2_-imbedded and regular PET yarns by microscope images. The black spots in Figure 3c are assumed to be Al, Sn, Sb and Ti, and in Figure 3d, they are Ti ingredients.

**Figure 4:**
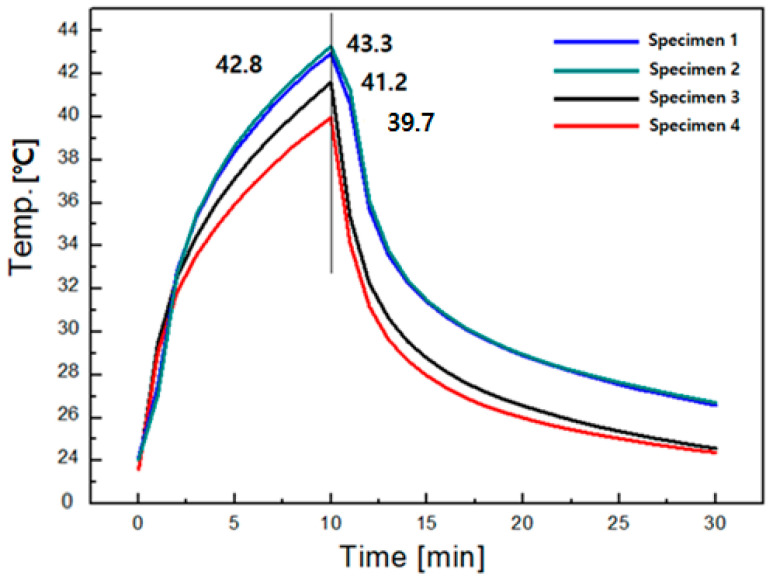
Light heat emission diagram of the fabric specimens.

The thermal radiation of the fabric specimens was assessed by a light heat emission experiment to examine how much heat they emit by the FIR emissivity according to the amount of the Al_2_O_3_/ATO particles imbedded in the yarns as the fabric specimens are exposed to the light. Figure 4 shows the light heat emission diagram of the four fabric specimens.

As shown in Figure 4, the maximum temperature of fabric specimens 1, 2 and 3 was higher than that of fabric specimen 4 due to the greater heat emission from the Al_2_O_3_/ATO/TiO_2_ particles imbedded in the yarns of fabric specimens 1, 2 and 3 than from TiO_2_ particles imbedded in the fabric specimen 4, which was verified by the FIR emission characteristics of the fabric specimens shown in Table 4.

Table 4 lists the FIR results of the constituent yarns of the fabric specimens. The difference of emission power between specimen 1 and 2 or 2 and 3 was 0.01 × 10^2^ W/m^2^·μm, but the difference between specimen 1 and 4 was 0.04 × 10^2^ W/m^2^·μm. It seems that the difference of emissive power according to wt. % of Al_2_O_3_/ATO particles is about 0.01 × 10^2^ W/m^2^·μm, which is relatively low compared with the difference between them and regular PET yarn specimen 4. The emissivity and emissive power of specimens 1, 2 and 3 were higher than those of specimen 4. This means that the Al_2_O_3_/ATO-imbedded PET fabrics exhibit higher effectiveness for the far-infrared emission than the TiO_2_-imbedded regular PET fabric does, resulting in a higher maximum temperature of the Al_2_O_3_/ATO-imbedded PET fabrics, as shown in Figure 4. This result was in accordance with the previous findings [2,3,4,5,6] related to the inorganic-particles-imbedded fabrics. Their findings [2,4,5] carried out using ZrC-imbedded fabrics exhibited higher maximum temperature of the ZrC-imbedded fabrics than that of the TiO_2_-imbedded regular PET fabric. Similar results were shown in the Al_2_O_3_-imbedded fabrics [3,6]. In addition, concerning the maximum temperature according to the Al_2_O_3_/ATO weight ratio of the core part among specimens 1, 2 and 3, the specimen with higher ATO weight percentage in the core of the yarn exhibited lower temperature, i.e., fabric specimen 3 than specimens 1 and 2, as shown in Figure 4. This was attributed to the heat shielding property of the ATO particles in the yarns, i.e., the thermal insulation property of the ATO enables it to shield the FIR emitted from the bulb of light heat emission apparatus, resulting in lower maximum temperature of fabric specimen 3 with high core ratio (70 wt.%) comparing with fabric specimens 1 and 2 with low core ratio (50 and 60 wt.% each). This was verified by the higher emissivity and emissive power of specimens 1 and 2 than those of specimen 3, as shown in Table 4, which resulted in a lower maximum temperature of fabric specimen 3 with high core ratio (70 wt.%) than that of fabric specimens 1 and 2 with core ratio of 50 and 60 wt.%. These results suggest that the higher inclusion (70%) of the ATO particles in the Al_2_O_3_/ATO/TiO_2_-imbedded yarns is less effective for heat release than the lower inclusion (50%) of the ATO particles because of the greater decrease in heat release caused by heat shielding of ATO particles in case of higher inclusion of ATO, i.e., even though the higher inclusion of the Al_2_O_3_ particles with higher wt.% of ATO enhances heat release, the heat shielding effect of the higher ATO particles is much more dominant than heat release effect of Al_2_O_3_, resulting in effective heat release through inclusion of the lower percentage of ATO.

### 3.2. UPF of the Al_2_O_3_/ATO/TiO_2_-Imbedded Fabrics

An understanding of how the UV protection of the Al_2_O_3_/ATO/TiO_2_-imbedded fabric is influenced by the wt. percentage of the Al_2_O_3_/ATO particles imbedded in the core is very important in relation to the heat release characteristics of the Al_2_O_3_/ATO/TiO_2_-imbedded fabrics. The UPF was measured and calculated as a measure of UV protection using an ATLAS M 284D, SDL apparatus.

**Figure 5:**
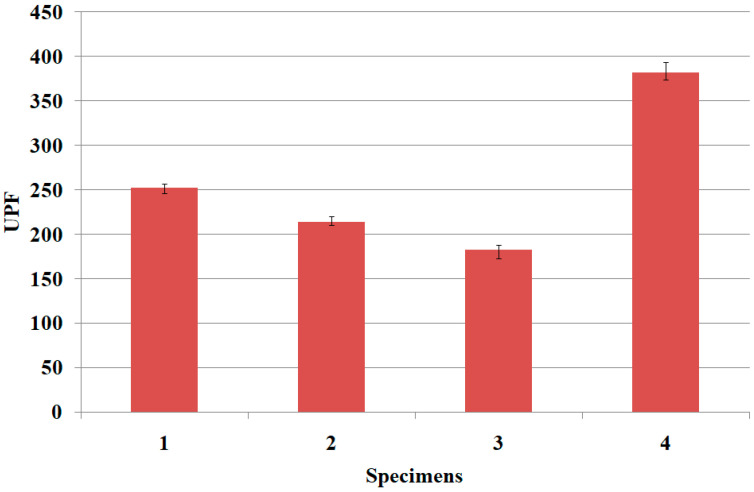
UPF of the fabric specimens.

Table 5 lists the UPF, rub-voltage and surface electrical resistivity of the four fabric specimens. The deviation in Table 5 is the difference between the maximum and minimum values of the experimental data. ANOVA (F-test) was carried out to verify the statistical significance of the experimental data shown in Table 5. Table 6 lists ANOVA data for the physical properties of the four fabric specimens. The mean value among the four fabric specimens for UPF, rub-voltage and SER was statistically significant, as F_0_ (V/V_e_) > F (3, 16, 0.95) and *p* < 0.05. Figure 5 presents the UPF of the fabric specimens.

As shown in Figure 5, the UPF of the TiO_2_-imbedded regular PET fabric (specimen 4) imbedded with TiO_2_ particles was the highest, followed by fabric specimens 1, 2 and 3. This means that the UV protection characteristic of the Al_2_O_3_/ATO inorganic particles imbedded in fabric specimens 1, 2 and 3 is inferior to that of the TiO_2_ inorganic particles imbedded in fabric specimens 4. This result was attributed to excellent absorbance over UV wavelength range (in particular UVA) of the TiO_2_ particles, i.e., this means that UV shielding/blocking/absorbing power of TiO_2_ particle is superior to that of the Al_2_O_3_ and ATO particles. According to previous studies [29,30,31], inorganic particles such as TiO_2_, ZnO, SiO_2_ and Al_2_O_3_, referred to “ceramics”, as being often preferred as UV blockers (scattering agents). Among them, TiO_2_ is the most effective UV blocker, which absorbs and scatters both UVA and UVB, and TiO_2_ reduces transmitted UV due to it reflecting and scattering UV radiation. This is assumed to be the reason for superior UPF of TiO_2_ particles.

In addition, the lower the wt. percentage of the Al_2_O_3_/ATO imbedded in the core part of the sheath/core yarns, the higher the UPF, which is because the lower the wt. percentage of Al_2_O_3_/ATO, the more TiO_2_ in the yarns, which makes it higher UPF. This result was similar to those previously reported [13,14,15,16], even though their results were for TiO_2_-coated fabrics, in which they reported the increased effectiveness of UV protection by coating treatment of the TiO_2_NPs on the fabric surface.

### 3.3. Anti-Static Property of the Al_2_O_3_/ATO-Imbedded Fabrics

**Figure 6:**
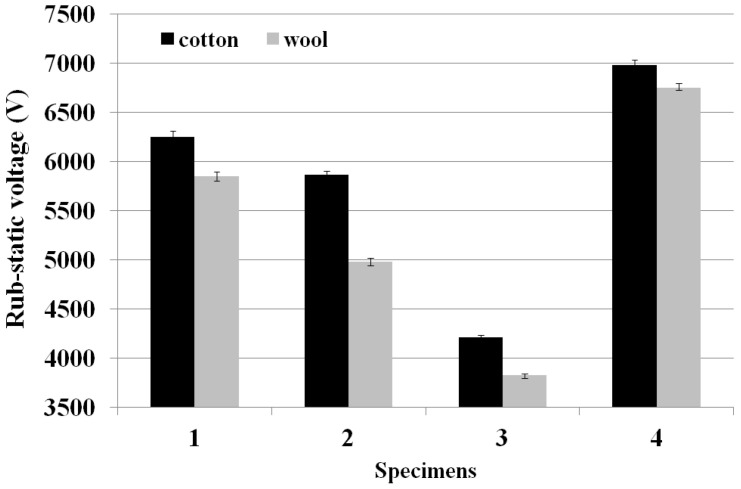
Rub-static voltage of the fabric specimens.

This study also examined the anti-static property of the Al_2_O_3_/ATO/TiO_2_-imbedded fabrics. Figure 6 presents the rub-static voltage of the Al_2_O_3_/ATO/TiO_2_-imbedded fabric specimens.

**Figure 7:**
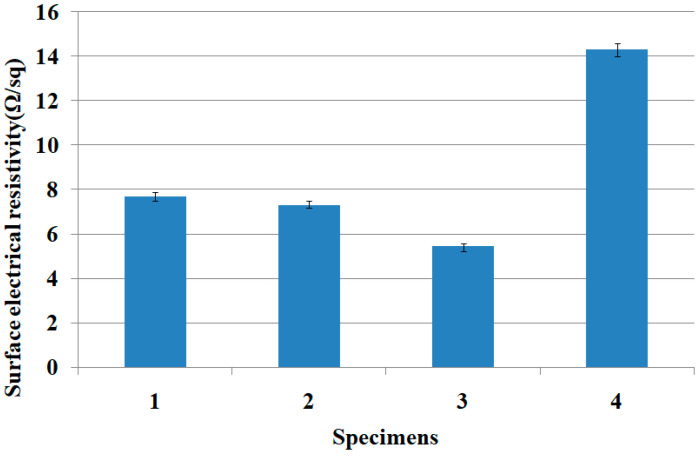
Surface electrical resistivity of the fabric specimens.

As shown in Figure 6, the rub-voltage of fabric specimen 3 (70 wt.% of Al_2_O_3_/ATO) for the cotton rubbing fabric was the lowest, followed by fabric specimens 2, 1 and regular TiO_2_-imbedded PET fabric, and a similar trend was found for the wool rubbing fabric. These results were attributed to the higher electrical conductivity of the ATO particles than that of the Al_2_O_3_ and TiO_2_ particles [12], i.e., ATO particles imbedded in the yarns help to effectively dissipate the static charge on the fabric surface due to higher electrical conductivity of the ATO than TiO_2_ and Al_2_O_3_ particles. According to the study [32] related to the physical and chemical structures of ATO for the conductivity of the ATO particles, the conductivity of the ATO depends on the antimony content and its oxidation state in the tin oxide lattice. While Sb^5+^ ions act as electron donors forming a shallow donor level close to the conduction band of SnO, Sb^3+^ behaves as an electron acceptor. If both oxidation states coexist, which is often observed for ATO materials, the resistivity is given by the ratio of Sb^5+^ and Sb^3+^ sites. These are the structural characteristics of the ATO with higher electrical conductivity than TiO_2_ and Al_2_O_3_ particles. These findings suggest that highly ATO-imbedded yarns enhance the anti-static property, i.e., Al_2_O_3_/ATO-imbedded yarn with high wt. percentage of Al_2_O_3_/ATO (specimen 3) has better electric conductivity than that of the TiO_2_-imbedded yarn (specimen 4), which results in lower rub-static voltage of fabric specimen 3, i.e., the static charge accumulated on the fabric surface is assumed to be dissipated due to the superior electrical conductive characteristic of the ATO inorganic particles imbedded in the yarns. Concerning the relationship between anti-static property and UPF of the Al_2_O_3_/ATO/TiO_2_-imbedded fabrics, according to a previous study [12], TiO_2_ and ZnO are commonly used as a UV blocker, and TiO_2__,_ ZnO and ATO provide anti-static effects. In this study, the Al_2_O_3_/ATO-imbedded fabric with lower wt.% of Al_2_O_3_/ATO exhibited a higher UPF and lower anti-static property (i.e., higher rub-static voltage); therefore, higher wt.% of ATO particles imbedded in the yarns is required for obtaining multi-functional fabrics with good UV cut and superior anti-static characteristics. This was verified by the surface electrical resistivity (SER) of the fabric specimens measured by the ACL 800 Megohmmeter, which was shown in Table 5. Figure 7 shows the SER (Ω/sq) of the Al_2_O_3_/ATO/TiO_2_-imbedded fabric specimens.

The SER of fabric specimen 3 was the lowest, which was in accordance with the result of the rub voltages of the fabric specimens. According to the previous studies [12,24,25], ATO-imbedded fabric and coated film exhibited excellent electric conductivity and high conducting property. In particular, the surface electrical resistivity of fabric specimens 1, 2, and 3 was much lower than that of fabric specimen 4. This means that the ATO particles in the yarns provide more effective electrical conductive characteristics than TiO_2_ imbedded in the yarn of fabric specimen 4. In addition, the superior anti-static property of the highly ATO-imbedded fabric, due to higher electrical conductivity, eliminates the flame by electric discharge occurring from friction between human body and garment during the wearing of clothing, i.e., protecting the wearer from electrostatic discharges, which is a critical point in workwear for protective clothing.

### 3.4. Tactile Hand Feel of Al_2_O_3_/ATO/TiO_2_-Imbedded Fabric Specimens

Inorganic particle imbedding treatment of the fabrics must not affect their desirable properties, such as tactile hand and wearing performance for fitness during the wearing of clothing. According to prior studies [33,34], tactile hand and wear comfort of flame-retardant/anti-static PET imbedded fabric were superior to regular cotton fabric [33], whereas the effects of functional finishing on the fabric’s tactile property were dependent on the types of finishes. In this study, how the Al_2_O_3_/ATO/TiO_2_ particles imbedded in the yarns affect the tactile hand feel and wearing performance of the fabric was investigated by the mechanical properties of the fabrics measured using the FAST system. Table 7 lists the mechanical properties of the fabric specimens.

**Figure 8:**
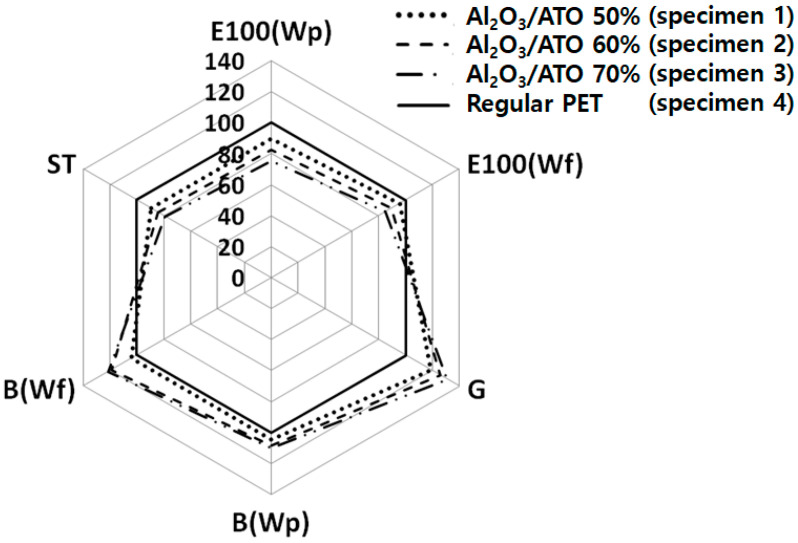
Diagram of the relative mechanical properties of the Al_2_O_3_/ATO-imbedded fabrics against a regular PET fabric.

Figure 8 presents the relative mechanical properties of the three Al_2_O_3_/ATO particle-imbedded fabrics to a TiO_2_-imbedded regular PET fabric. The extensibility (E100), bending rigidity (B), shear modulus (G) and compressibility (ST) of the three Al_2_O_3_/ATO-imbedded fabric specimens were plotted as a ratio to those of the TiO_2_-imbedded regular PET fabric specimen.

**Figure 9:**
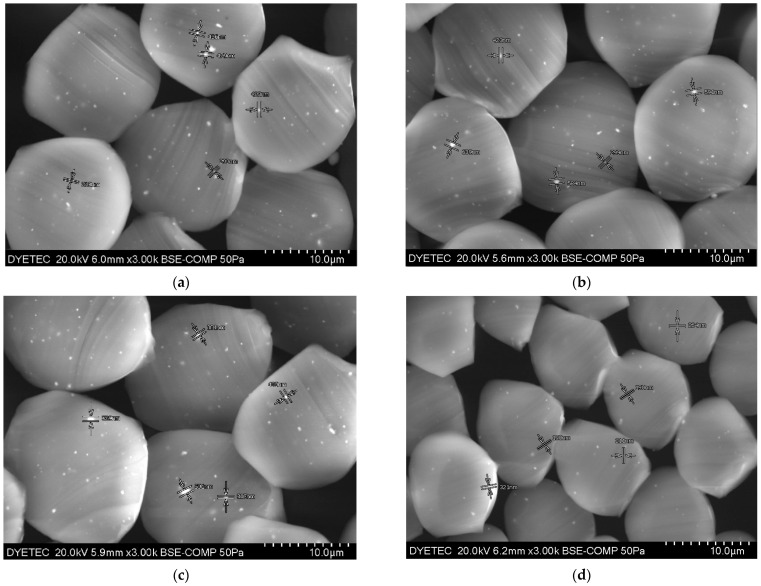
SEM (×3000) images of four yarn specimens. ((**a**) Specimen 1, (**b**) Specimen 2, (**c**) Specimen 3, (**d**) Specimen 4).

As shown in Figure 8, the extensibilities (E100) in the warp and weft directions of the Al_2_O_3_/ATO-imbedded fabrics (specimens 1, 2 and 3) were lower than those of the TiO_2_ -imbedded regular PET fabric (specimen 4), respectively. In addition, the compressibilities (ST) of fabric specimens 1, 2 and 3 were lower than those of the fabric specimen 4. This was attributed to the Al_2_O_3_/ATO particles imbedded in the yarns, i.e., the longitudinal and compressional deformations of the fabric may be prohibited by the Al_2_O_3_/ATO particles in the yarns, resulting in lower extensibility and compressibility. Furthermore, fabric specimen 3 with higher wt. percentage of the Al_2_O_3_/ATO particles in the core region exhibited lower extensibility and compressibility than fabric specimens 1 and 2 did, resulting in it being less extensible and compressible. On the other hand, the bending rigidities (B) in the warp and weft directions of the Al_2_O_3_/ATO-imbedded fabrics (specimens 1, 2 and 3) were higher than those of the TiO_2_-imbedded regular PET fabric (specimen 4), respectively, which was caused by the longer deflection due to the larger inorganic particles (Al_2_O_3_/ATO) than TiO_2_ imbedded in the yarns, resulting in a higher bending rigidity, as estimated in Equation (3). In particular, the shear modulus (G) of fabric specimens 1, 2 and 3 was higher than that of the regular PET fabric specimen 4. This means that the Al_2_O_3_/ATO-imbedded fabrics are stiffer than the TiO_2_-imbedded regular PET fabric is, which is also attributed to the larger Al_2_O_3_/ATO particles than TiO_2_ imbedded in the yarns, which may be protected from the in-plane deformation of the extension and shear of the fabrics, resulting in low extensibility and high shear modulus. In addition, the bending rigidity and shear modulus of fabric specimen 3 were higher than those of fabric specimens 1 and 2, which was due to higher wt. percentage of the Al_2_O_3_/ATO particles imbedded in the core region of the yarns of fabric specimen 3. Figure 9 shows the SEM images of the yarn cross-sections of the fabric specimens. As shown in Figure 9a–d, more white spots were observed in (a), (b) and (c) than in (d), which imparted lower extensibility and compressibility with higher bending rigidity and shear modulus to the Al_2_O_3_/ATO-imbedded fabrics than those of the TiO_2_-imbedded regular PET fabric.

Based on these mechanical properties, Al_2_O_3_/ATO particles imbedded in the yarns were assumed to impart an uncomfortable tactile hand feel and wear fitness to the Al_2_O_3_/ATO particle-imbedded fabrics compared to the TiO_2_-imbedded regular PET one, which showed a somewhat different result compared with a previous study [33]. Furthermore, among the three Al_2_O_3_/ATO-imbedded fabrics, the fabric with lower wt. percentage of Al_2_O_3_/ATO imbedded in the core of the yarn exhibited more comfortable tactile hand feel and wear fitness than the fabric with higher wt. percentage of the Al_2_O_3_/ATO particles, which is similar to prior result [34] depending on the types of finishes.

## 4. Conclusions

This study examined the UV cut and anti-static properties with heat release by FIR emissivity of the Al_2_O_3_/ATO/TiO_2_-imbedded PET fabrics with different wt. percentages of Al_2_O_3_/ATO particles in the sheath/core yarns. The lower inclusion of ATO particles in the Al_2_O_3_/ATO/TiO_2_-imbedded yarns was more effective for heat release than the higher inclusion of the ATO particles due to the heat shielding property of the ATO particles in the yarns. In addition, the fabric with lower wt. percentage of Al_2_O_3_/ATO exhibited higher UPF due to more TiO_2_ particles in the fabric with lower wt.%, indicating that contribution to UPF of the TiO_2_ particles imbedded in the yarn is superior to that of the Al_2_O_3_ and ATO particles. The fabric with higher wt. percentage of Al_2_O_3_/ATO exhibited lower rub-static voltage due to its higher electric conductivity than that of the PET fabric with lower wt. percentage of Al_2_O_3_/ATO, i.e., highly ATO-imbedded yarn enhanced the anti-static property with much lower SER, which means that ATO inorganic particles imbedded in the yarns provide superior anti-static property to the Al_2_O_3_ and TiO_2_ particles. On the other hand, Al_2_O_3_/ATO particles imbedded in the yarns imparted an uncomfortable tactile hand feel and wear fitness to the Al_2_O_3_/ATO-imbedded fabric. Based on multi-functional characteristics for warm-up suit fabric, Al_2_O_3_/ATO-imbedded sheath/core yarns with 50 wt.% are of practical use for engineering woven fabrics with excellent heat release, UV cut and tactile hand feeling, whereas, considering superior anti-static property, Al_2_O_3_/ATO-imbedded yarns with 70 wt.% (i.e., highly ATO-imbedded) are applicable to warm-up suit for winter with good heat release and excellent anti-static property.

## Figures and Tables

**Table 1:** Mixing ratio of M/B chip in bi-component melt spinning.

	PET Chip (kg)	M/B Chip Weight (kg)		Mixed Polymer (kg)	Mixing Ratio wt.% of M/B		
		Al_2_O_3_	ATO		Al_2_O_3_	ATO	Total (wt.%)
Concentration of M/B chip (wt.%)	-	20 (wt.%)	20 (wt.%)				
Al_2_O_3_/ATO PET	47.25	2	0.75	50	0.8	0.3	1.1

Note: M/B: master batch.

**Table 2:** Spinning condition in the conjugated melt spinning equipment.

	Characteristics	Spinning Conditions		
Spinning temp.	Spinbeam temp. (°C)	287		
	Mainfold temp. (°C)	290/290		
	Extruder heating temp. (°C) (S/C)	300–305/301–304		
Roller speed and temp.	First GR speed (m/min)	1446		
	Second GR speed (m/min)	3910		
	First GR temp. (°C)	85		
	Second GR temp. (°C)	105		
	F/R speed (m/min)	4000		
Yarn specification	Yarn linear density (d/f)	SDY 50d/24f		
	Sheath/core weight ratio (%)	50:50	40:60	30:70

Note: S/C: sheath/core, GR: godet roller, F/R: reed roller, d/f: denier/filaments.

**Table 3:** Specifications of four fabric specimens.

Specimen No.	Yarn Count (d/f)		Sheath: Core (S/C) Ratio	Fabric Density (Picks, Ends/In)		Thickness	Weight
	Wp	Wf		Wp	Wf	(mm)	(g/m^2^)
1	PET 50/72	Al_2_O_3_/ATO	5:5	154	100	0.13	85.4
2	PET 50/72	S/C PET	4:6	154	100	0.12	84.8
3	PET 50/72	50/24	3:7	154	100	0.13	85.6
4	PET 50/72	Reg PET 50/72	-	154	100	0.12	84.2

**Table 4:** Emissivity and emissive power of the four fabric specimens.

Specimens	Specimen 1		Specimen 2		Specimen 3		Specimen 4	
	Mean	Dev.	Mean	Dev.	Mean	Dev.	Mean	Dev.
Emissivity	0.885	0.1 × 10^−3^	0.884	0.2 × 10^−3^	0.882	0.1 × 10^−3^	0.866	0.2 × 10^−3^
Emissive power (W/m^2^·µm)	3.44 × 10^2^	0.001 × 10^2^	3.43 × 10^2^	0.002 × 10^2^	3.42 × 10^2^	0.001 × 10^2^	3.40 × 10^2^	0.001 × 10^3^

Note: dev. = max. − min.

**Table 5:** Physical properties of the fabric specimens.

Specimens		UPF		Rub-Voltage (V)				Surface Electrical Resistivity (Ω/sq)	
		Mean	Dev.	Cotton		Wool			
				Mean	Dev.	Mean	Dev.	Mean	Dev.
1	Al_2_O_3_/ATO 50% PET	252.5	11.0	6250	99	5850	93	7.70 × 10^10^	0.4 × 10^10^
2	Al_2_O_3_/ATO 60% PET	214.2	10.0	5860	77	4980	74	7.30 × 10^10^	0.3 × 10^10^
3	Al_2_O_3_/ATO 70% PET	182.5	15.5	4210	44	3820	51	5.44 × 10^10^	0.4 × 10^10^
4	TiO_2_-imbedded regular PET	382.3	19.3	6980	111	6750	68	14.30 × 10^10^	0.6 × 10^10^

Note: dev. = max. − min.

**Table 6:** ANOVA data for the physical properties of the four fabric specimens.

Physical Properties		F-Value (F_0_)	F (3, 16, 0.95)	*p*-Value
UPF		1801.5	3.24	1.92 × 10^–20^
Rub-voltage	Cotton	6042.3	3.24	1.22 × 10^–24^
	Wool	9768.9	3.24	2.62 × 10^–26^
SER		2836.0	3.24	5.14 × 10^–22^

**Table 7:** Mechanical properties of the fabric specimens.

Specimens		E 100 (%)		G (N/m)	B (µN·m)		ST (mm)
		Wp	Wf		Wp	Wf	
1	Al_2_O_3_/ATO 50% PET	0.87	0.45	52.0	31.3	34.5	0.017
2	Al_2_O_3_/ATO 60% PET	0.80	0.42	55.1	32.5	39.5	0.016
3	Al_2_O_3_/ATO 70% PET	0.73	0.40	57.4	33.0	40.2	0.015
4	TiO_2_-imbedded regular PET	0.97	0.47	43.8	30.3	33.2	0.019

## Data Availability

The data presented in this study are available on request from the author.
